# Irritability is Common and is Related to Poorer Psychosocial Outcomes in Youth with Functional Abdominal Pain Disorders (FAPD)

**DOI:** 10.3390/children5040052

**Published:** 2018-04-19

**Authors:** Sarah Nelson, Erin Moorman, Michael Farrell, Natoshia Cunningham

**Affiliations:** 1 Department of Anesthesia, Pain and Perioperative Medicine, Boston Children’s Hospital, Boston, MA 02115, USA; 2 Department of Psychiatry, Harvard Medical School, Boston, MA 02215, USA; 3 Division of Behavioral Medicine and Clinical Psychology, Cincinnati Children’s Hospital Medical Center, Cincinnati, OH 45229, USA; erin.moorman@cchmc.org (E.M.); natoshia.cunningham@cchmc.org (N.C.); 4 Division of Pediatric Gastroenterology, Hepatology, and Nutrition, Cincinnati Children’s Hospital Medical Center, Cincinnati, OH 45229, USA; Michael.farrell@cchmc.org; 5 Department of Pediatrics, University of Cincinnati College of Medicine, Cincinnati, OH 45267, USA

**Keywords:** pediatric, functional abdominal pain, emotion reactivity, gender differences

## Abstract

Functional abdominal pain disorders (FAPD) are associated with increased emotional problems which, in turn, exacerbate functional impairment. However, irritability, which relates both to internalizing and externalizing problems, has not been specifically examined in these youths. Irritability may be common and adversely impact functioning in pediatric FAPD, particularly for males who are more likely to experience such symptoms. The current study examined the relationship between irritability and psychosocial and pain-related impairment in youth with FAPD. Data were gathered as part of a larger study examining a psychological treatment for youth with FAPD and were compared to previously published data on irritability in healthy controls and in youth with severe emotional dysregulation. For the current study, participants (ages 9–14) with FAPD and caregivers completed measures of child irritability, pain-related and psychosocial functioning, and parent functioning. Pearson correlations revealed significant positive associations between irritability and anxiety, depressive symptoms, pain catastrophizing, and caregiver distress. Results also indicated that parents reported significantly greater irritability in males, but males and females reported similar rates of irritability. Gender moderated the relationship between child-report of irritability and anxiety only. Future research may include tailoring of behavioral intervention approaches for pediatric FAPD to specifically target symptoms of irritability.

## 1. Introduction

Functional abdominal pain disorders (FAPD) affect up to 12% of children and adolescents between the ages of 4 and 18 [1,2,3]. Research in adult populations indicates that FAPD (e.g., irritable bowel syndrome subtype) is associated with significant psychosocial impairment and mood problems, including symptoms of anxiety such as increased worries, [4], symptoms of depression such as increased feelings of sadness, loss of interest/pleasure, etc. [5], and difficulty with regulating emotions, manifested as increased irritability [4,6,7]. In youth with FAPD, significant research suggests that symptoms of anxiety and depression (i.e., internalizing symptoms) are frequently observed [2,8,9] and associated with increased pain-related impairment (e.g., decreased physical and academic functioning [2,10]). While the literature generally suggests comparable rates of externalizing problems between youth with FAPD and healthy controls [11,12,13,14], it is unknown if specific symptoms that may manifest in both internalizing (i.e., mood) and externalizing (i.e., behavioral) disorders, such as irritability which may be evident through increased anger, becoming easily annoyed, losing temper easily, etc. [15]), uniquely impact youth with pediatric FAPD, or if differences emerge by gender. Irritability, in particular, may be an important construct to examine as 1) it is a hallmark symptom of both internalizing disorders, such as Major Depressive Disorder (MDD), and externalizing disorders such as Oppositional Defiant Disorder (ODD) and 2) more recently has also been demarcated as an important symptom in relation to the newer mood diagnosis of Disruptive Mood Dysregulation Disorder (DMDD; characterized by significantly irritable or angry mood and frequent temper displays, etc.; Diagnostic and Statistical Manual of Mental Disorders, 5th edition (DSM-V [16])).

Further examination of the prevalence of irritability symptoms as a proxy for both externalizing symptoms and mood dysregulation may be acutely relevant to youth with FAPD, given the high rates of psychological comorbidities that may involve or be associated with mood symptoms that may manifest as irritability, including anxiety and depressive symptoms [9,17], in this population. Recent findings suggest that the presence of psychological problems that are related to irritability such as anxiety [18] can significantly and negatively impact psychosocial treatment outcomes for pediatric FAPD [19], though no one has systematically investigated the unique role of irritability in relation to functioning in youth with FAPD. Further, youth with FAPD and other recurrent pain syndromes may experience increased parent/caregiver stress within the family system [20,21], in addition to the added stress of coping with their medical condition, which may in turn increase irritability and magnify pain-related disability.

Although a risk categorization system incorporating child reports of anxiety, pain levels, and disability has been developed to identify youth with FAPD who are at risk for persistent disability [8], the psychosocial and emotional factors like irritability that may underlie the relationship between FAPD and increased pain-related and psychosocial impairment remain poorly understood. It may be that presence of irritability, which has been found to be associated with a variety of issues including anxiety, depression, and emotion regulation difficulties [4,17,22,23], accounts for clinical impairment in a subset of youth with FAPD and additional risk factors such as high levels of anxiety.

Further, it may be important to understand irritability from both patient and parent perspectives, given that youth may underreport such symptoms due to perceived stigma (social desirability response bias) or other social factors [24,25], similar to what is observed when children are asked to report on their own externalizing symptoms, and often underreport such symptoms as compared with their caregiver [26]. Furthermore, research on irritability in non-pain populations also indicates that there are gender differences in self-report. Specifically, males may report higher rates of irritability and associated externalizing symptoms [27] as opposed to anxiety or depression, when compared to females [22,28]. However, gender differences in rates of irritability have not been explicitly examined in FAPD. Learning more about the incidence and associated characteristics of increased irritability in youth with FAPD, in addition to specific variations based on gender, may serve to enhance understanding of which youth may be at increased risk for poor outcomes and may benefit from a tailored psychosocial intervention.

The current study aimed to (1) examine rates of irritability in youth with FAPD and (2) investigate how increased irritability may relate to psychosocial and pain-related outcomes. It was hypothesized that (1) increased irritability will be common in youth with FAPD and (2) increased irritability would be significantly associated with greater psychological, family-related, and pain-related impairment in functioning. (3) Based on the adult literature, it was also hypothesized that males would experience higher rates of irritability than females.

## 2. Materials and Methods

### 2.1. Participants

Participants included youth with FAPD between the ages of 9–14 presenting for treatment at one of several pediatric gastroenterology clinics at a children’s hospital. During the screening process for study eligibility, a trained research coordinator had the referring physician complete a checklist based on Rome IV criteria [29]. Furthermore, at each patient’s baseline visit, they were administered a comprehensive functional gastrointestinal disorders (FGID) interview based on the Rome III [30] by a trained postdoctoral fellow or clinical research coordinator. Based on this interview, it was confirmed that all participants met criteria for FAPD. These data were also compared to previously published data on irritability in healthy controls and in youth with severe emotional dysregulation [15], due to this measure’s lack of use in other pediatric pain populations. The current study is approved by the Cincinnati Children’s Hospital Medical Center (CCHMC) IRB (IRB # 2015-1388; Date of Approval: 9 March 2015).

### 2.2. Procedures

Data were gathered as part of a larger study examining the effect of a psychological intervention to target pain and co-occurring anxiety in youth with FAPD. Data were collected (between 2015 and 2017) in person by a trained clinical research coordinator during a pediatric gastroenterology office visit at Cincinnati Children’s Hospital Medical Center in Cincinnati, OH. All study procedures were approved by the hospital Institutional Review Board. After receipt of informed consent and assent for participation of both children and a primary caregiver (to complete questionnaires about their own/child’s functioning and engage in the psychological intervention), youth were asked to complete screening questionnaires (i.e., Functional Disability Inventory; FDI) to determine eligibility for the primary study. If eligible for the primary study (more than minimal score of >7 on the FDI for two weeks or greater and a physician-confirmed diagnosis of FAPD), participants and their caregivers were invited to complete a baseline assessment, where measures of parent- and child-reported child irritability, pain-related impairment, and psychosocial impairment were obtained. Parents also completed a measure of their own distress.

#### 2.2.1. Questionnaires

##### 2.2.1.1. Background and Demographics

Participants’ caregivers were asked to answer questions regarding general demographics including child age and race, and parent/caregiver educational attainment.

##### 2.2.1.2. FGID Status

The Rome III Diagnostic Questionnaire for Functional Gastrointestinal Disorders [30] assesses symptoms of FGIDs, including functional abdominal pain. Participants’ were administered the questionnaire in clinical interview format. Responses were scored according to the pediatric Rome III criteria.

##### 2.2.1.3. Irritability Measure

###### Affective Reactivity Index (ARI, Parent and Child Report)

The Affective Reactivity Index is a validated measure of irritability for ages 5–17 [15]. For both parent and child report, respondents are asked to rate the child’s level of irritability on six items (e.g., “gets angry easily, “often loses his/her temper”) based on the past week. Items are rated on a 0–2 scale (0 = “not true at all”; 1 = “somewhat true”; 2 = “certainly true”). The total score consists of the sum of six items, with higher scores indicative of greater presence of irritability. The internal consistency of the ARI for the current sample were excellent for both child-report (0.90) and for parent-report (0.92).

##### 2.2.1.4. Measures of Psychological, Family-Related and Pain-Related Functioning

###### Functional Disability Index (FDI, Parent and Child Report)

The FDI, a self-report questionnaire, has been validated for use in youth chronic pain populations between the ages of 8 and 17 and is used to assess difficulty in completing various activities due to health symptoms [31,32]. Available responses for each of the 15 items range from 0 (no trouble) to 4 (impossible). Item responses are summed to create a total disability score (range = 0–60) which is interpreted as follows: no/minimal disability = 0–12; moderate disability = 13–29; severe disability = 30+. The internal consistency of the FDI for the current sample was 0.83, which is considered very good.

###### Pain Catastrophizing Scale (PCS, Child Report)

The child version of the PCS contains 13 items related to thoughts and feelings about pain experienced by the child. Response items range from not at all (0), mildly (1), moderately (2), severely (3), and extremely (4). Total scores range from 0–52, with higher scores reflecting greater catastrophizing. Total catastrophizing scores were used for analyses in this study. The PCS-C has been validated in pediatric pain samples between the ages of 8 and 16 [33]. The internal consistency of the PCS for the current sample was excellent (α = 0.93).

###### Child Depression Inventory (CDI-2, Child Report)

The Child Depression Inventory (CDI-2) [34] is a 28-item self-report questionnaire that assesses symptoms of depression in children and adolescents. It has been consistently validated for use in children/adolescents between the ages of 7 and 17. Items, scored on a 3-point scale, are summed to derive a total score, with higher scores indicating greater severity of depressive symptoms (range 0–56). Of note, for the purposes of this study, the two overlapping irritability items on the CDI-2 were removed (e.g., “I feel cranky…”, etc.) in order to minimize overlap with the irritability measure. The internal consistency of the CDI-2 for the current sample is 0.90, which is considered excellent.

###### Screen for Child Anxiety-Related Disorders (SCARED, Parent and Child Report)

The SCARED is a widely used screening instrument for clinically significant anxiety symptoms in youth [35]. It has 41 items, is based on the Diagnostic and Statistical Manual of Mental Disorders (DSM-IV-TR), and has been validated for use in children ages 8–18 [36]. The SCARED has been validated in a pediatric pain sample [37] and in clinical samples of youth with abdominal pain conditions [1,38,39]. Youth are asked to report frequency of anxiety symptoms over the past three months. Responses include: “not true”, “sometimes true”, and “often true”. Total scores range from 0 to 82, with higher scores reflecting greater levels of anxiety. The internal consistency of the SCARED for the current sample was excellent (α = 0.93).

###### Depression Anxiety Stress Scales (DASS-21, Parent Report)

The DASS-21 assesses symptoms of depression, anxiety and stress in adults (parents) using a 21-item questionnaire [40,41]. Each item is rated on a 4-point Likert scale ranging from 0 (did not apply to me) to 3 (applied to me very much or most of the time). The internal consistency of the DASS for the current sample was excellent, at 0.93.

#### 2.2.2. Statistical Analyses

Data were analyzed using SPSS v.23 [42]. Measures of central tendency and variability were performed for all study measures using visual inspection. Internal consistency reliability for each study questionnaire was examined by utilizing the reliability analyses function in SPSS. These results pertaining to irritability rates were then compared to prior validation samples as detailed in the participants section above using two one-way ANOVAs (for child- and parent-report) with post hoc testing (Tukey HSD) to examine individual group differences. In order to examine the relationship between parent- and child-report of irritability and pain-related (e.g., functional disability), psychosocial (e.g., anxiety and depressive symptoms, pain catastrophizing), and family-related (e.g., parent-functioning) outcomes, Pearson product moment correlations were performed.

Next, gender differences in irritability levels were explored. First, independent samples t-tests were performed with gender as the grouping variable and irritability (separately for parent- and child-report) as the dependent variables. Following this, data were separated by gender and Pearson product moment correlations were performed again to examine the association between irritability in each of the above identified outcomes. Lastly, for any significant associations that were found among one gender but not the other, gender was explored as a moderator (separately for parent and child report) of the relationship between irritability and the identified clinical outcome using hierarchical linear regression. In the first step of each regression, gender and either the parent-report or child-report of irritability were included as the independent variables (IVs), with the interaction term (gender x parent-report/child-report of irritability) included in the second step. A False Discovery Rate (FDR; [43]) Type 1 error control was used for all analyses. Specifically, three separate sets of analyses were conducted to obtain Benjamini–Hochberg values for the full sample, and then for males and females separately [43]. All *p* values cited in the current study are Benjamini–Hochberg *p* values.

## 3. Results

### 3.1. Participant Characteristics

Participants included 69 youth (26 males, 43 females) between the ages of 9 and 14 (mean age = 11.5). The current sample population was predominantly Caucasian (89.9%), which aligns with previous research in pediatric chronic pain samples [38]. A minority of the sample was males (37.7%), which is consistent with previous studies in similar populations [13,44]. At baseline, participants reported a mean pain intensity of 3.4 (on a 0–10 scale) and a mean FDI score of 18.3 (moderate disability). Through visual inspection, it was found that no norms of central tendency or variability were violated for any variables of interest. Please see Table 1 for additional information on sociodemographic characteristics of the sample.

### 3.2. Rates of Irritability in Youth with FAPD vs. Validation Samples

**Figure 1:**
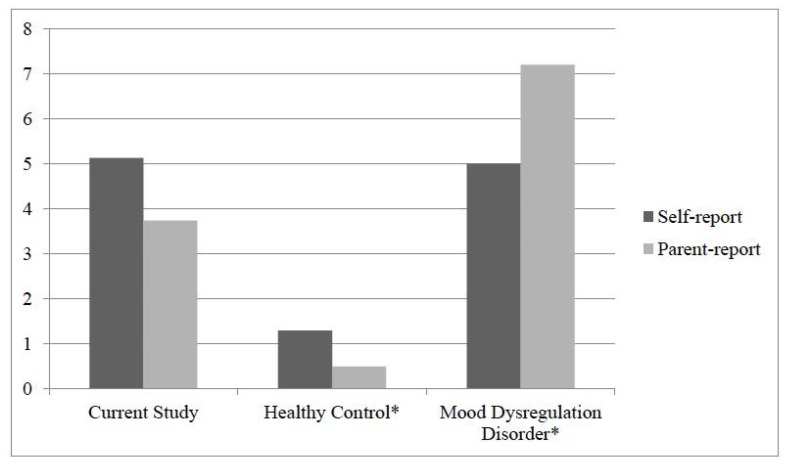
Mean rates of irritability in the current sample when compared to validation samples. * Data are utilized from original validation study, Stringaris et al., 2012.

When compared to validation samples [15], results of the one-way ANOVA indicated significant group differences (F (2, 335) = 64.43, *p* < 0.001) with youth with FAPD (mean irritability = 5.1) reporting significantly higher levels of irritability than (psychologically) healthy youth (mean irritability = 1.2; Difference = −3.87, 95% CI = −4.85 to −2.89, *p* < 0.001) and comparable levels of irritability to youth with severe mood dysregulation disorders (mean irritability = 4.6; Difference = −0.52, 95% CI = −1.52 to 0.48, *p* = 0.44 ). One-way ANOVA results for parent-report of child irritability also indicated significant group differences (F (2, 335) = 203.8, *p* < 0.001) with, based on the parent perspective, youth with FAPD (mean irritability = 3.7) experiencing significantly higher levels of irritability when compared to (psychologically) healthy youth (mean irritability = 0.43; Difference = −3.27, 95% CI = −4.21 to −2.33, *p* < 0.001) and significantly lower levels of irritability when compared to youth with severe mood dysregulation disorders (mean irritability = 7.2; Difference = 3.48, 95% CI = 2.52 to 4.44, *p* < 0.001). Please see Figure 1 for a graphical depiction of these results.

### 3.3. Irritability in Relation to other Psychosocial and Pain-Related Outcomes

Pearson product moment correlations were performed to examine the overall association between child- and parent-report of youth irritability with pain-related (i.e., functional disability), psychosocial (i.e., anxiety, depressive symptoms), and family-related (i.e., parent functioning) outcomes. These analyses revealed moderate correlations between parent- and child-report of irritability (*r_pearson_* = 0.484, *p* < 0.001); however, parent- and child-report of irritability differentially related to clinical outcomes. For child-reported irritability, these analyses revealed significant positive associations with child-report of their own anxiety, (child) pain catastrophizing, and (child) depressive symptoms. No significant associations were found between child-report of irritability and functional disability (either parent- or child-report) or any caregiver distress outcomes (i.e., stress, anxiety, depression). For parent-reported irritability, results revealed significant positive associations with child-report of their own anxiety, child depressive symptoms, and parent/caregiver stress, parent/caregiver anxiety, and parent/caregiver depressive symptoms, no significant associations were found between parent-report of irritability, functional disability (either parent- or child-report), or parent-report of child anxiety, or child pain catastrophizing. Please see Table 2 for complete details.

### 3.4. Irritability by Gender

Significant gender differences in irritability were revealed for parent-report of irritability (*t* (64) = −2.168, *p* = 0.036), with males displaying higher levels of irritability as compared to females (M_males_ = 5.12; M_females_ = 2.85). No significant differences were found between genders on child-report of irritability (M_males_ = 5.35; M_females_ = 5.00).

When the relationship between pain-related and psychosocial outcomes were separately examined by gender in males, child-report of irritability was found to be significantly associated with higher levels of (child) anxiety (*r_pearson_* = 0.593, *p* = 0.006), (child) depressive symptoms (*r_pearson_* = 0.627, *p* = 0.006), and pain catastrophizing (*r_pearson_* = 0.482, *p* = 0.044). No significant correlations were found between child-report of irritability and any parent-reported outcomes (i.e., parent-report of child anxiety, parent-report of functional disability, parent functioning items) or child functional disability. No significant correlations were found between parent-report of irritability and any child-reported (i.e., child anxiety, child depressive symptoms, pain catastrophizing, functional disability) or parent-reported (child anxiety, functional disability, parent/caregiver distress) outcomes, aside from parent-report of child anxiety (*r_pearson_* = 0.533, *p* = 0.021), parent/caregiver stress (*r_pearson_* = 0.587, *p* = 0.011), and parent/caregiver depressive symptoms (*r_pearson_* = 0.517, *p* = 0.025), which were found to be significant. Please see Table 3 for complete details on these analyses.

In females, significant correlations were found between child- and parent-report of irritability and (child) depressive symptoms (child-report: *r_pearson_* = 0.574, *p* < 0.001; parent-report: *r_pearson_* = 0.524, *p* = 0.009). No significant correlations were found between irritability and child anxiety (either parent- or child-report) or any pain-related or family-related outcomes

### 3.5. Moderator Analyses

Outcomes found to be associated with parent- and child-report of irritability in one gender but not the other (i.e., child-report of anxiety) were examined in a separate model with each outcome as the dependent variable (DV). The results of the hierarchical linear regression model with child anxiety as the outcome indicated that the inclusion of the interaction term (gender x child-report of irritability) accounted for a significant amount of the variance in child anxiety (Δ*R^2^ =* 0.059, Δ*F* (3, 65) = 5.069, *p* < 0.001, t (68) = 2.251, *p* = 0.28). Full details of these analyses are included in Table 4.

**Figure 2:**
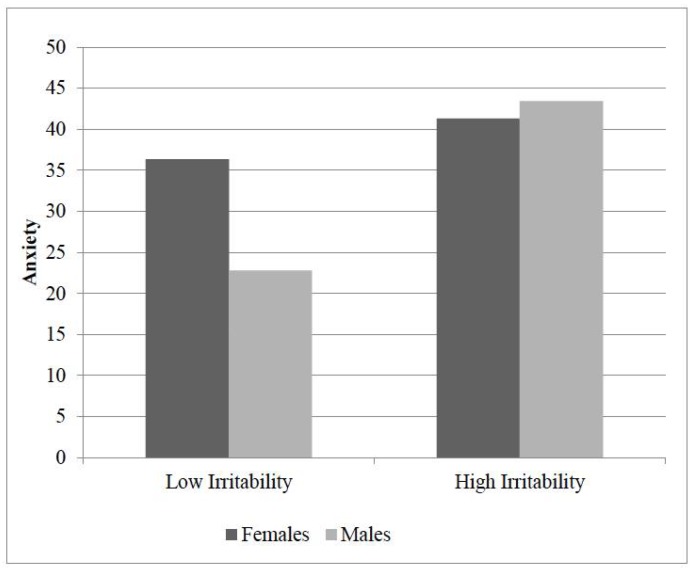
Graphical representation of the interaction of gender between child-report of irritability and anxiety.

To further examine this effect, separate post hoc linear regression analyses were employed for males vs. females. Results of these analyses revealed that as child-reported irritability increases, (child-report of) child anxiety increases in males only (*R^2^* = 0.35, *F* (1, 24) = 13.013, *p* = 0.001, *t* (25) = 3.607). Please see Figure 2 for a graphical representation of these results.

## 4. Discussion

This is the first study to our knowledge that examines the incidence of increased irritability and its association with psychosocial and pain-related impairment in a pediatric chronic pain population. Irritability has been previously found to be increased in adults with FAPD and is associated with poorer psychosocial and pain-related outcomes, such as increased issues with mood/anxiety or greater disability [6,7]. The current study’s preliminary results expand upon these findings by examining the rates of irritability in pediatric FAPD, and in exploring the relationship between heightened irritability and psychosocial and pain-related outcomes. Study findings suggest that individuals (and perhaps males in particular) with FAPD may struggle with increased irritability that corresponds to poorer global functioning. This is important because while the majority of youth with FAPD are female, a subset are male [13,44]. These results imply that males who are at increased risk for both pain-related and psychosocial impairment may have unique clinical profiles characterized by increased irritability, and as such, may have specific treatment needs geared towards targeting such symptoms. Interestingly, comparison to validation samples also indicates that youth with FAPD report comparable levels of irritability with youth who have been diagnosed with significant mood dysregulation issues such as bipolar disorder. This is of particular relevance as evidence suggests that youth with FAPD may struggle significantly in several areas of their daily lives including social/interpersonal and academic functioning, as observed in youth with severe mood regulation issues [45,46].

Consistent with our study hypothesis, parent-report of child irritability did reveal that males with FAPD experience significantly higher rates of irritability than females, which confirms the importance of obtaining a parent-report of such symptoms versus a child-report where such symptoms may be minimized. Results from the current study also suggest that, unlike child anxiety which tends to be better captured with a child-report [39], the parent-reported measure of irritability may be more sensitive/clinically meaningful for males [47]. This is similar to studies reporting increased rates of externalizing behaviors (such as higher rates of reported irritably in males than females when assessing for oppositional defiant disorder) via parent-report than child-report [26]. Given that irritability may be a component of mood (i.e., anxiety) and behavioral problems, it may be that both parent- and child-report of symptoms are important to gather in order to get a more comprehensive picture of psychological functioning [39,48]. These findings also suggest that parents experience increased distress when male children with FAPD display increased irritability in contrast to females where internalizing issues such as anxiety or depressive symptoms are more commonly reported [49]. More research is needed on the variations in parental response to youth distress in pediatric FAPD and other chronic pain conditions.

Further, correlational analyses revealed that child-reported irritability in males was significantly associated with greater psychosocial impairment in youth, including higher rates of anxiety, depressive symptoms, and pain catastrophizing, while irritability in females was only associated with increased depressive symptoms. Caregiver distress (e.g., anxiety, depressive symptoms, stress) was also notably associated with increased irritability in males only (no significant associations were found in females). In order to expand upon these findings, gender was specifically examined as a moderator in the relationship between irritability and psychosocial impairment in youth and parent outcomes. Significant moderation was found between child-reported irritability and anxiety, indicating that as irritability rates in males with FAPD increase, rates of anxiety increase as well. This is particularly notable given how detrimental the presence of anxiety can be on general pain-related and psychosocial functioning [38,39,50] as well as on psychological treatment outcomes [19] in youth with chronic pain. It is also consistent with research in other youth populations indicating a strong connection between irritability and anxiety symptoms [51].

In addition to anxiety, results generally suggest that the presence of increased irritability in males is significantly more impactful on child well-being (e.g., depression, pain catastrophizing) than when females report or exhibit elevated irritability. Interestingly, while females tend to have higher rates of anxiety and depression when compared to males in general populations (across multiple cultures) [49], males’ psychosocial distress in conjunction with FAPD (and more broadly) may be better expressed by constructs such as irritability, which is also a core feature of clinical externalizing disorders, such as oppositional defiant disorder [16] and major mood disturbance such as disruptive mood dysregulation disorder [16]. Assessing for constructs such as irritability in conjunction with more commonly assessed for symptoms of anxiety or depression may also capture a greater number of youth in distress, given that a subset of youth with (and without) chronic pain tend to underreport symptoms of irritability due to perceived stigma [24].

Strengths of the current study include the recruitment and analysis of a fairly heterogeneous sample of youth with FAPD. With almost 38% of these youths identifying as male, the current sample is more representative of community samples than other clinical studies that have over-represented females (e.g., 80% or more female sample, etc.) [13,38]. This will likely increase generalizability of the current results. Further, this study’s recruitment methods of integrated screening during a child’s regularly scheduled gastroenterology visit may have allowed researchers to gain access to the more diverse array of male and female study participants. Future research should examine these recruitment methods in other pediatric pain settings in order to gain a greater understanding of the most effective methods for examining diverse populations. 

Despite the significant strengths of this study, limitations are also present which should be considered when interpreting the results. The sample size for the current study was fairly small and from a single geographic area (Midwest region of the United States). Similarly, participant ages were limited to ages 9–14 due to the current study being part of a larger trial examining a new psychological therapy for youth of that age range. Furthermore, we felt it was important to examine psychosocial outcomes for younger individuals to potentially inform efforts at preventing the development of more significant psychopathology as youth age. However, we recognize that this limits the generalizability of study results to other age groups, such as older teens. We plan to examine more diverse age groups in future studies. Further, the current sample consisted of youth who were seeking medical/psychological treatment and were only admitted into the study after meeting a minimum threshold for functional disability. As such, the psychosocial and/or pain-related impairment that they reported may be elevated when compared to non-treatment seeking community samples. The current study was also limited by using a single parent/child report of irritability. Utilizing other measures of irritability (e.g., behavioral observation) may help enhance future research. Finally, the use of cross-sectional data limits the current study’s findings with respect to generalizability to long-term outcomes. As such, future research should include longitudinal data on relevant psychosocial and pain-related outcomes in youth with FAPD.

Due to the significant effect that increased irritability may have on relevant psychosocial outcomes, exploring this phenomenon in future research in other chronic pain populations may be of particular relevance. It may also be particularly important to examine differences in irritability between males and females with varying chronic pain conditions in order to confirm or provide greater insight into the associations between irritability and psychosocial impairment in males specifically that were found in the current study. Finally, given that irritability has been examined in the literature as relating directly to the manifestation of certain psychological issues (e.g., anxiety, depression) [18,52], and that males in this study specifically experienced concomitantly higher rates of irritability and anxiety, incorporating its consideration into treatment may significantly aid in achieving positive outcomes [53]. Specifically, the development and testing of behavioral interventions that address emotional problems such as irritability which may manifest in broader internalizing/externalizing issues and also foster greater parent efficacy (e.g., parent–child interaction therapy (PCIT [54]) as part of a pain coping skills program, particularly for males with FAPD, may bolster outcomes in these youths. To inform such research, we plan to explore the role of irritability in predicting treatment outcomes for youth who completed a tailored cognitive behavioral intervention to target pain and anxiety in our future work.

## 5. Conclusions

The results of this study are the first to examine the rates and correlates of issues with irritability both in a general population of youth with FAPD and by gender. Increased irritability was associated with greater psychosocial impairment. Further, increased irritability in males with FAPD was associated with greater psychosocial impairment when compared to females with FAPD. Future research should continue to examine these constructs in larger populations with varying types of chronic pain conditions.

## Figures and Tables

**Table 1:** Sample Demographics (*n* = 69).

Child Characteristics	*n* (%)
Age, mean (SD)	11.5 (1.7)
Male	26 (37.7)
Race	
White	62 (89.9)
African American	1 (1.4)
Asian	1 (1.4)
Native Hawaiian/Other Pacific Islander	0 (0)
American Indian	1 (1.4)
Biracial	4 (5.8)
	**mean (SD)**
Pain Intensity	3.4 (1.9)
FDI	18.3 (8.4)
CDI	14.8 (9.2)
PCS	26.9 (11.7)
SCARED	36.1 (15.9)
ARI	5.1 (4.2)
**Parent/Caregiver Characteristics**	***n* (%)**
Male	8 (11.6)
Mother Education Level	
High School	13 (18.8)
Some College/Technical School	21 (30.4)
College Degree	23 (33.3)
Graduate Degree	12 (17.4)
Father Education Level	
Less than High School	5 (7.2)
High School	22 (31.9)
Some College/Technical School	19 (27.5)
College Degree	17 (24.6)
Graduate Degree	6 (8.7)
	**mean (SD)**
FDI, parent report	14.4 (11.4)
SCARED, parent report	31.2 (14.4)
ARI, parent report	3.7 (4.0)
DASS Stress	6.5 (4.4)
DASS Anxiety	3.2 (4.1)
DASS Depression	3.2 (3.9)

FDI = Functional Disability Inventory; CDI = Child Depression Inventory, Second edition; PCS = Pain Catastrophizing Scale; SCARED = Screen for Child Anxiety Related Disorders; ARI = Affective Reactivity Index; DASS = Depression Anxiety Stress Scales.

**Table 2:** Association between parent- and child-reported irritability and psychosocial and pain-related outcomes in the overall sample.

	1	2	3	4	5	6	7	8	9	10	11
1. ARI Self-report	1										
2. ARI Parent-report	0.484 **	1									
3. SCARED Self-report	0.391 **	0.127 *	1								
4. SCARED Parent-report	0.263	0.351	0.553 **	1							
5. FDI Self-report	0.197	0.251	0.164	0.138	1						
6. FDI Parent-report	0.088	0.177	0.107	0.349 **	0.476 **	1					
7. CDI	0.582 **	0.342 *	0.554 **	0.242	0.295 *	0.091	1				
8. PCS	0.307 *	0.098	0.404 **	0.175	0.276 *	0.190	0.512 **	1			
9. DASS Stress	0.123	0.513 **	0.119	0.375	0.218	0.231	0.044	0.024	1		
10. DASS Anxiety	0.128	0.364 **	0.145	0.398 **	0.203	0.312 *	0.045	−0.011	0.647 **	1	
11. DASS Depression	−0.068	0.329 *	0.064	0.363	0.148	0.339 **	−0.027	−0.002	0.597 **	0.702 **	1

Note: * correlation is significant at the 0.05 level (2-tailed); ** Correlation is significant at the 0.01 level (2-tailed); ARI = Affective Reactivity Index; SCARED = Screen for Anxiety and Related Disorders; FDI = Functional Disability Inventory; CDI = Child Depression Inventory 2; PCS = Pain Catastrophizing Scale; DASS = Depression and Anxiety Scales; False Discovery Rate (FDR) Type-1 error control was used for all comparisons.

**Table 3:** Association between parent- and child-reported irritability and psychosocial and pain-related outcomes separately for females (top) and males (bottom).

	1	2	3	4	5	6	7	8	9	10	11
1. ARI Self-report	1	0.567 **	0.252	0.242	0.197	0.169	0.574 **	0.249	0.093	0.195	−0.047
2. ARI Parent-report	0.411	1	0.144	0.227	0.125	0.152	0.524 **	0.029	0.282	0.233	0.094
3. SCARED Self-report	0.593 **	0.212	1	0.430 *	0.283	0.173	0.575 **	0.450 *	0.093	0.131	0.005
4. SCARED Parent-report	0.297	0.533 *	0.691 **	1	0.096	0.322	0.317	0.209	0.204	0.291	0.144
5. FDI Self-report	0.198	0.283	0.124	0.189	1	0.423 *	0.342	0.252	0.001	0.253	0.144
6. FDI Parent-report	−0.066	0.194	0.063	0.403	0.559 *	1	0.170	0.285	0.041	0.298	0.250
7. CDI	0.627 **	0.269	0.519 *	0.159	0.305	0.008	1	0.434 *	0.078	0.189	0.028
8. PCS	0.482 *	0.403	0.315	0.130	0.465	0.085	0.613 **	1	−0.010	0.041	0.054
9. DASS Stress	0.139	0.587 *	0.254	0.596 **	0.271	0.431	0.096	0.292	1	0.393	0.476 *
10. DASS Anxiety	0.048	0.405	0.218	0.529 *	0.121	0.340	−0.031	0.048	0.773 **	1	0.522 **
11. DASS Depression	−0.110	0.517 *	0.152	0.611 **	0.122	0.466	−0.058	0.004	0.705 **	0.849 **	1

Note: * Correlation is significant at the 0.05 level (2-tailed); ** Correlation is significant at the 0.01 level (2-tailed); ARI = Affective Reactivity Index; SCARED = Screen for Anxiety and Related Disorders; FDI = Functional Disability Inventory; CDI = Child Depression Inventory; PCS = Pain Catastrophizing Scale; DASS = Depression and Anxiety Stress Scales; A False Discovery Rate Type-1 error control was used for all comparisons.

**Table 4:** Multiple regression analyses examining the interaction effect of gender on the relationship between child-reported irritability and anxiety.

Model 1	b	SE	β	*t*	*p*
Gender ^	−5.690	3.637	−0.174	−1.565	0.122
Irritability *	1.502	0.420	0.398	3.578	0.001
*R^2^ =* 0.183, *F* (2, 66) = 7.415, *p* = 0.001					
Model 2					
Gender ^	−15.581	5.636	−0.477	−2.765	0.007
Irritability *	0.793	0.515	0.210	1.541	0.128
Irritability *-by-Gender	1.896	0.842	0.437	2.251	0.028
Δ*R^2^ =* 0.059, Δ*F* (3, 65) = 5.069, *p* < 0.001					

^ Gender is coded dichotomously (0 = females; 1 = males); * Child-report of irritability as measured by the Affective Reactivity Index (ARI).
